# The role of brain natriuretic peptide in atrial fibrillation: a substudy of the Substrate Modification with Aggressive Blood Pressure Control for Atrial Fibrillation (SMAC-AF) trial

**DOI:** 10.1186/s12872-021-02254-5

**Published:** 2021-09-16

**Authors:** Willy Weng, Rajin Choudhury, John Sapp, Anthony Tang, Jeff S. Healey, Isabelle Nault, Lena Rivard, Isabelle Greiss, Jordan Bernick, Ratika Parkash

**Affiliations:** 1 University of Ottawa Heart Institute, Ottawa, ON Canada; 2 Dalhousie University, 1796 Summer Street, Rm 2501-D, Halifax Infirmary, Halifax, NS B3H 3A7 Canada; 3 Western University, London, ON Canada; 4 McMaster University, Hamilton, ON Canada; 5 Université Laval, Laval, QC Canada; 6 Montreal Heart Institute, Montreal, QC Canada; 7 Centre Hospitalier de L’Université de Montréal, Montreal, QC Canada

**Keywords:** Atrial fibrillation, Catheter ablation, Recurrence, Biomarker

## Abstract

**Background:**

Catheter ablation is an established therapy for atrial fibrillation but is limited by recurrence; efforts have been made to identify biomarkers that predict recurrence. We investigated the effect of baseline NT-proBNP on AF recurrence following catheter ablation in patients randomized to aggressive (< 120/80 mmHg) or standard blood pressure management (< 140/90 mmHg) in the Substrate Modification with Aggressive Blood Pressure Control trial (SMAC-AF).

**Methods:**

The SMAC-AF study included 173 patients resistant or intolerant to at least one class I or III antiarrhythmic drug. We studied the effect of baseline NT-proBNP on the primary outcome of AF recurrence > 3 months post-ablation.

**Results:**

Of the 173 patients, 88 were randomized to the aggressive cohort, and 85 into the standard group. The primary outcome occurred in 61.4% of those in the aggressive arm, versus 61.2% in the standard arm. In the aggressive group, logNT-proBNP predicted recurrence (HR 1.28, *p* = 0.04, adjusted HR 1.43, *p* = 0.03), while in the standard cohort, it did not (HR 0.94, *p* = 0.62, adjusted HR 0.83, *p* = 0.22). NT-proBNP ≥ 280 pg/mL also predicted occurrence in the aggressive (HR 1.98, *p* = 0.02) but not the standard cohort (HR 1.00, *p* = 1.00).

**Conclusion:**

We conclude that pre-ablation NT-proBNP may be useful in predicting recurrence in hypertensive patients and identifying patients who benefit from aggressive blood control and upstream therapies.

*Trial registration*: NCT00438113, registered February 21, 2007.

## Introduction

Radiofrequency catheter ablation has been established as a valuable therapeutic option for atrial fibrillation (AF), although high rates of recurrence after ablation are a limitation. Factors predicting recurrence include type of AF (paroxysmal versus persistent), ejection fraction, structural heart disease and hypertension [1, 2]. The recent Substrate Modification with Aggressive Blood Pressure Control (SMAC-AF) randomized control trial did not find that aggressive blood pressure control prior to ablation had an effect on recurrence rates of AF post ablation [3]. Atrial fibrosis and remodeling is thought to play a role in predisposition to AF [4], and there has been developing interest in identifying biomarkers associated with fibrosis that may predict response to therapy for AF [5–7]. Brain natriuretic peptide (NT-proBNP), is commonly used to guide clinical decision in heart failure management, and has been studied as a biomarker [8, 9]. NT-proBNP is released by myocytes in response to adverse hemodynamic conditions [10], and is also associated with cardiac fibrosis [11]. Thus, it has been theorized that NT-proBNP levels may be reflective of underlying predisposition to AF, as well as being a marker of atrial fibrosis remodeling. Pre-ablation NT-proBNP has been shown in some populations to be a predictor of recurrence post-ablation [12–14].

The purpose of this study was to explore whether NT-proBNP is useful as a marker of recurrence post-ablation in hypertensive patients. NT-proBNP has not been shown to be elevated in those with hypertension without cardiac dysfunction [15]. Furthermore, the randomized nature of SMAC-AF allows us to investigate how the association of NT-proBNP and outcomes differed with the effect of the additional blood pressure management.

## Methods

This is a secondary analysis of the SMAC-AF study, the details of which have been published previously (NCT00438113). [3] In brief, 173 hypertensive patients (defined as B*p* ≥ 130/80 mmHg), resistant or intolerant to at least one class I or III antiarrhythmic drug, and scheduled to undergo catheter ablation, were randomized to undergo aggressive blood pressure management (target of 120/80 mmHg) or standard management (target of 140/90 mmHg), in the time leading up to ablation (0–6 months), and throughout study follow-up. The primary outcome was time to symptomatic AF, atrial tachycardia, or atrial flutter lasting more than 30 s, 3 months after ablation. The study took place from July 2011 to October 2015; the median follow-up was 14.0 months. The protocol of the original study was approved by the local ethics review board at all participating institutions (QEII Health Sciences Centre, Halifax, NS; Centre Hospitalier Universitaire de Montreal, Montreal Heart Institute, Montreal, QC; Centre Hospitalier Universitaire de Sherbrooke, Sherbrooke, QC; London Health Sciences Center, London, ON; Quebec Heart Institute, Quebec City, QC; Hamilton Health Sciences, Hamilton, ON; Royal Jubilee Hospital, Victoria, BC; University of Ottawa Heart Institute, Ottawa, ON; Toronto General Hospital, St Michael’s Hospital, Toronto, ON; Libin Cardiovascular Institute of Alberta, Mazankowski Alberta Heart Institute, Edmonton, AB.) All methods were carried out in accordance with relevant guidelines and regulations.

### Ablation protocol

All patients underwent pulmonary vein isolation with either cryoballoon therapy or radiofrequency ablation. All ablations were performed within 0–3 months of randomization. Antiarrhythmic medications were discontinued 5 half-lives prior to the ablation with the exception of amiodarone, which was discontinued for four weeks prior to ablation. Peri-procedural anticoagulation was instituted in all patients for one month prior to the procedure, and for three months post procedure. Long-term anticoagulation was left to the discretion of the investigator. Three-dimensional maps of the left atrium were constructed with the use of a nonfluoroscopic navigation system (CARTO, Biosense Webster, Diamond Bar, CA, USA, or ESI, St. Jude Medical, St. Paul, Minnesota, USA). Continuous wide-area circumferential lesions were created encircling right and left pulmonary venous ostia guided by electroanatomic mapping with an irrigated, cooled-tip radiofrequency ablation catheter with pulmonary vein isolation being the endpoint. A maximum temperature of 40 °C with maximum power of 40 W was to be delivered, except for the posterior wall, where the power was not to exceed 25 W. Lesions were complete when electrogram amplitude was reduced by ≥ 80% of baseline. Additional non-pulmonary vein ablation was left to the discretion of the operator, including complex fractional atrial electrogram ablation and additional linear ablation.

### Serum markers

NT-proBNP and C-reactive protein (CRP) levels were pre-specified in the trial protocol as a secondary outcome. The biomarker levels were measured at time of randomization, as well as 12 months after randomization, using a standard CRP and NT-proBNP assay (Cobas, Roche Diagnostics GmbH). These assays reliably measure NT-proBNP concentrations from 0.6–4130 g/mL, and CRP concentrations between 0.3 and 350 mg/L.

### Data collection and follow-up

Baseline clinical characteristics were measured at time of randomization. Patients were seen in clinic with a 12-lead electrocardiogram at 3 months, 6 months and every 6 months thereafter for a maximum of 24 months. Recurrence of AF was documented using transtelephonic monitoring, performed routinely twice weekly for 2 weeks, then every 3 months for the duration of the study until a primary outcome was reached. The primary outcome was atrial fibrillation, atrial tachycardia or atrial flutter (atrial cycle length ≥ 220 ms) lasting longer than 30 s and was adjudicated by a blinded committee.

### Analysis

Baseline characteristics are described as mean (standard deviation) or median (first quartile, third quartile) for continuous variables, and count (percent) for categorical variables, where appropriate. Comparisons between groups were made using t-tests for continuous variables, and chi-square tests for categorical variables. We examined for correlation between baseline logNT-proBNP and baseline characteristics, using Pearson coefficient method for continuous variables, and chi-square for categorical variables.

To investigate the effect of baseline NT-proBNP on the primary outcome, a Cox proportional hazard model was used.. A receiver operator curve was used to determine a stratification level for NT-proBNP; a cutoff of 280 pg/mL was found to have maximum area-under-curve. Kaplan–Meier product-limit estimates were used to compare those with elevated NT-proBNP (≥ 280 pg/mL) and without (< 280 pg/mL); hazard ratios were calculated using a Cox proportional model. This was repeated for those in the aggressive and standard pressure control cohorts. Adjusted analysis was performed, using baseline characteristics that were clinically relevant, including sex and age, as well as those characteristics that differed between the two groups determined by p-value of < 0.10 in univariable testing, in a multivariable Cox proportional hazard model. Change in NT-proBNP from baseline to 12 months was also tested using Cox proportional hazards modeling on the primary outcome.

## Results

There were 173 patients included for analysis, 88 of whom were randomized to the aggressive cohort, and 85 to the standard group. The baseline characteristics for these patients is shown in Table 1. The majority of the patients had a pre-existing diagnosis of hypertension; the remainder had a documented blood pressure ≥ 130/80 mmHg to gain entry into the study.

**Table 1:** Baseline characteristics are shown in the cohort, and in patients with NT-proBNP measured, separated by treatment group

Variable:	Overall (n = 173)	Standard group (n = 75)			Aggressive group (n = 78)		
		NT-proBNP ≥ 280^*^ (n = 24)	NT-proBNP < 280 (n = 51)	*p* value	NT-proBNP ≥ 280 (n = 25)	NT-proBNP < 280 (n = 53)	*p* value
Age (years, mean ± SD)	59.7 ± 8.7	62.7 ± 6.8	57.5 ± 9.9	0.02	62.2 ± 7.4	58.7 ± 8.8	0.09
Women n (%)	45 (26.0)	9 (37.5)	12 (23.5)	0.21	10 (40)	9 (17)	0.03
Type of AF n (%):				0.09			0.06
Persistent	74 (42.8)	10 (41.7)	32 (63)		11 (44)	35 (66)	
Paroxysmal	99 (57.2)	14 (58.3)	19 (37.3)		14 (56)	18 (34)	
AF duration (months, mean ± SD)	57.2 ± 71.5	42.2 ± 35.6	65.5 ± 79.8	0.12	67.3 ± 99.8	51.1 ± 58.7	0.52
Systolic Blood Pressure (mmHg, mean ± SD)	142.6 ± 12.0	144.0 ± 16.6	141.5 ± 11.0	0.50	145.5 ± 11.8	141.3 ± 10.7	0.13
Hypertension n (%)	130 (75.0)	20 (83.3)	36 (70.6)	0.24	18 (72.0)	42 (79.3)	0.50
Diabetes n (%)	22 (12.7)	2 (8.3)	7 (13.7)	0.49	3 (12)	8 (15.1)	0.71
CHADS2 n (%):				0.61			0.73
0	39 (22.5)	3 (12.5)	13 (25.5)		7 (28)	11 (20.8)	
1	108 (62.4)	18 (75.0)	31 (60.8)		13 (52)	33 (62.3)	
2	18 (10.4)	2 (8.3)	5 (9.8)		3 (12)	7 (13.2)	
> 2	8 (4.6)	1 (4.2)	2 (3.9)		2 (8)	2 (3.8)	
LVEF (%, mean ± SD)	58.6 ± 8.2	58.3 ± 9.4	60.1 ± 6.1	0.43	55.9 ± 12.1	59.7 ± 7.6	0.19

Characteristics are compared between those with NT-proBNP ≥ 280 pg/mL and < 280 pg/mL, within each treatment group. The aggressive cohort had a target blood pressure of < 120/80 mmHg, and the standard cohort had a target of < 140/90 mmHg

AF, atrial fibrillation; SBP, systolic blood pressure; LVEF, left ventricular ejection fraction

^*^pg/mL

The primary outcome occurred in 61.4% of those in the aggressive arm, versus 61.2% in the standard arm (*p* = 0.76). NT-proBNP data was available for 153 patients (88.4%). There was no difference in baseline NT-proBNP (*p* = 0.73), LVEF (*p* = 0.72), age (*p* = 0.70), sex (*p* = 0.51), AF duration (*p* = 0.14) or baseline systolic blood pressure (*p* = 0.67) between aggressive and standard groups. AF type, mean blood pressure and number of antihypertensives agents were similar at baseline. Patients in the aggressive blood pressure arm were treated for a median of 3.5 months (interquartile range, 2.5–4.2 months) before ablation; the standard blood pressure arm received usual care for a similar duration (median of 3.1 months, interquartile range 2.6–4.2 months, *p* = 0.578). At time of ablation, patients in the aggressive arm were on more antihypertensives (4.61 vs 3.00, *p* < 0.0001). At 6 months, compared to the standard cohort, the aggressive cohort had lower blood pressure (systolic 123.2 ± 13.2 mmHg vs 135.4 ± 15.7 mmHg, diastolic 76.7 ± 11.4 mmHg vs 80.8 ± 10.2 mmHg, *p* < 0.001).

### NT-proBNP and baseline characteristics

There was a correlation between logNT-proBNP and age (r = 0.43, *p* < 0.0001), LVEF (r = 0.26, *p* = 0.003). LogNT-proBNP was higher in those with persistent AF (*p* = 0.0018). There was no relationship found between logNT-proBNP and pre-randomization AF duration (*p* = 0.43), LA size (*p* = 0.54), systolic blood pressure (SBP) (*p* = 0.17). Women had higher NT-proBNP levels: 38.8% of those with NT-proBNP ≥ 280 pg/mL were female, compared with 20.2% of those with NT-proBNP < 280 pg/mL (*p* = 0.01).

### NT-proBNP and outcomes

The correlation between baseline logNT-proBNP and outcomes is shown in Table 2. In the aggressive blood pressure cohort, baseline logNT-proBNP predicted recurrence (HR 1.28, *p* = 0.04), while in the standard cohort, it did not (HR 0.94, *p* = 0.62). After controlling for age, sex, LVEF, baseline SBP, and AF type, logNT-proBNP was still a predictor in the aggressive group (HR 1.43, *p* = 0.03), but not in the standard group (HR 0.83, *p* = 0.22) (Table 3), or in the entire study cohort (HR 1.04, *p* = 0.74). The test for interaction between treatment arm and baseline logNT-proBNP on the primary outcome demonstrated a trend to statistical significance (*p* = 0.07).

**Table 2:** Univariable predictors of post-ablation recurrence of atrial fibrillation are shown

	Aggressive		Standard		Whole cohort	
	HR (95% CI)	P value	HR (95% CI)	P value	HR (95% CI)	P value
Age (years)	0.99 (0.96–1.03)	0.69	**1.03** (**1.00–1.07)**	**0.04**	1.01 (0.99–1.03)	0.35
Sex (Female)	1.17 (0.63–2.18)	0.62	1.4 (0.78–2.5)	0.26	1.29 (0.84–1.96)	0.25
Persistent AF	1.06 (0.62–1.81)	0.83	0.75 (0.43–1.3)	0.3	1.13 (0.77–1.66)	0.54
Duration of AF	1.00 (0.99–1.01)	0.81	1.00 (1.00–1.01)	0.07	1.00 (1.00–1.01)	0.29
Baseline SBP (mmHg)	**1.03** (**1.00–1.05)**	**0.02**	1.01 (0.99–1.03)	0.38	**1.02** (**1.00–1.03)**	**0.02**
Baseline SBP ≥ 140 mmHg	**2.14** (**1.22–3.73)**	**0.01**	0.83 (0.48–1.43)	0.49	0.76 (0.51–1.11)	0.15
Diabetes	0.76 (0.34–1.67)	0.49	1.58 (0.71–3.51)	0.26	1.04 (0.59–1.82)	0.89
LA Size	1 (0.95–1.05)	0.91	0.99 (0.94–1.04)	0.74	0.99 (0.96–1.03)	0.74
Baseline NT-proBNP ≥ 280 (pg/ml)	**1.98** (**1.13–3.51)**	**0.02**	1.00 (0.55–1.83)	0.99	1.40 (0.92–2.12)	0.10
Log-NT-proBNP (pg/ml)	**1.28** (**1.01–1.62)**	**0.04**	0.94 (0.74–1.20)	0.62	1.09 (0.92–1.29)	0.31
CRP	1.00 (0.95–1.05)	0.89	0.99 (0.92–1.07)	0.82	1.00 (0.96–1.04)	0.81

Significant results (*p* < 0.05) are bolded

All characteristics were measured at time of randomization

AF, atrial fibrillation; SBP, systolic blood pressure; LVEF, left ventricular ejection fraction; LA, left atrium; CRP, C-reactive protein

**Table 3:** Multivariable predictor predictors of post-ablation recurrence of atrial fibrillation are shown, by blood pressure treatment group, with corresponding adjusted hazard ratios

Predictor	Aggressive cohort (n = 88)		Standard cohort (n = 85)	
	HR (95% CI)	*p* value	HR (95% CI)	*p* value
logNT-proBNP	**1.43 (1.03–1.99)**	**0.03**	0.83 (0.61–1.12)	0.22
Age	0.96 (0.92–1.01)	0.12	**1.05 (1.01–1.10)**	**0.01**
Sex	0.90 (0.40–2.00	0.79	1.08 (0.47–2.45)	0.86
SBP	**1.03 (1.00–1.05)**	**0.045**	1.01 (0.98–1.04)	0.48
LVEF	1.02 (0.98–1.06)	0.35	1.00 (0.95–1.06)	0.94
AF type	0.95 (0.48–1.88)	0.87	1.04 (0.53–2.05)	0.89

Significant results (*p* < 0.05) are bolded

All characteristics were measured at time of randomization. The aggressive cohort had a target blood pressure of < 120/80 mmHg, and the standard cohort had a target of < 140/90 mmHg

AF, atrial fibrillation; SBP, systolic blood pressure; LVEF, left ventricular ejection fraction

**Fig. 1:**
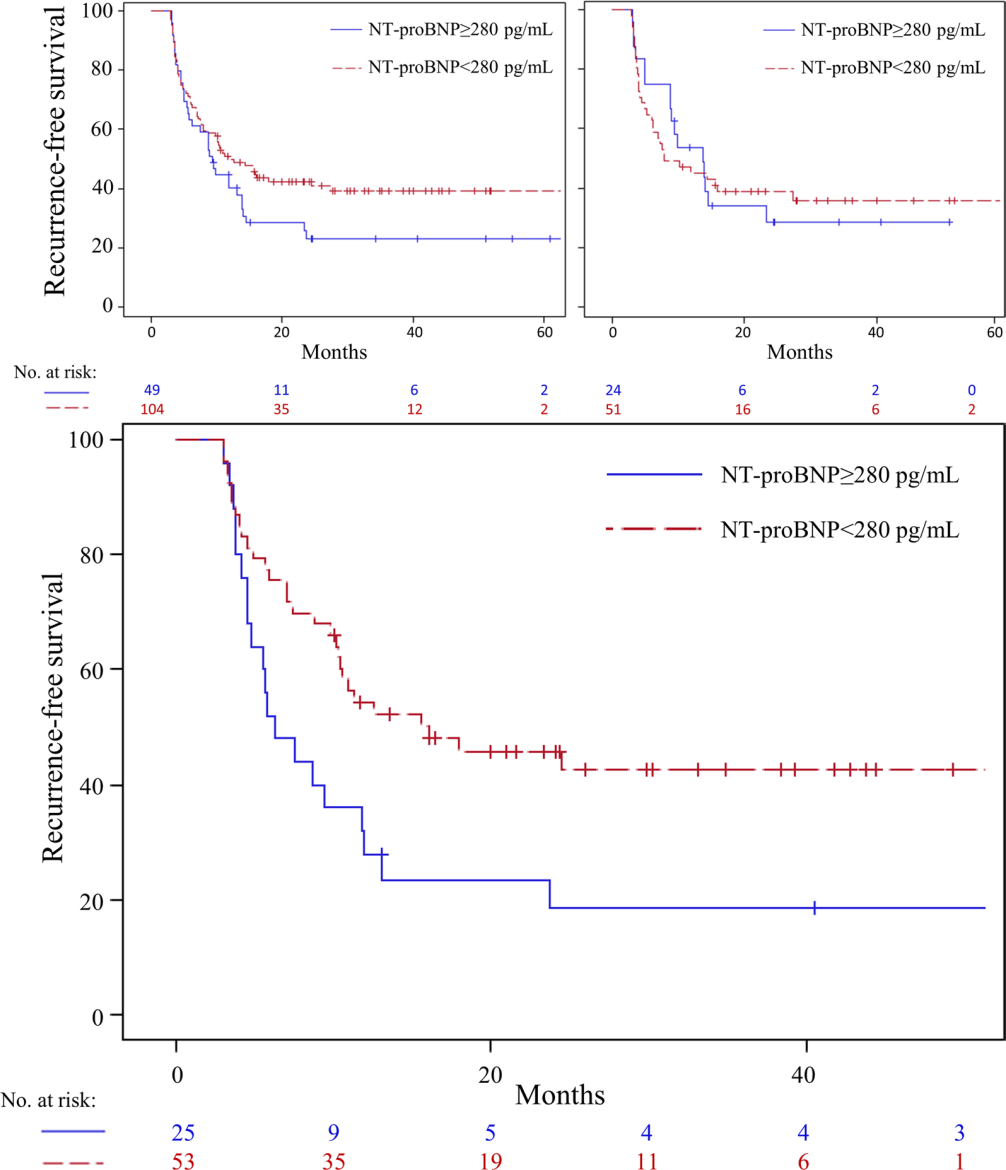
NT-proBNP as predictor of recurrence after atrial fibrillation ablation. Atrial fibrillation recurrence-free survival after ablation, stratified by baseline NT-proBNP ≥ 280 pg/mL and < 280 pg/mL. Top left: among the overall cohort (n = 173), there was no difference in recurrence between the groups (for NT-proBNP ≥ 280 pg/mL, HR 1.40, 95%CI 0.92–2.12, *p* = 0.10). Top right: among patients (n = 85) undergoing standard blood pressure management, < 140/90 mmHg, there was no difference (for NT-proBNP ≥ 280 pg/mL, HR 1.00, 95%CI 0.55–1.83, *p* = 0.998). Bottom: among patients (n = 88) undergoing aggressive blood pressure management, < 120/80 mmHg, those with baseline NT-proBNP ≥ 280 pg/mL had increased recurrence (HR 1.98, 95%CI 1.13–3.51, *p* = 0.02)

Stratifying by baseline NT-proBNP using a cutoff of 280 pg/mL, there was no difference in AF recurrence in the entire cohort (HR 1.40, *p* = 0.11) (Fig. 1). In the aggressive group, those with NT-proBNP ≥ 280 pg/mL had increased recurrence (HR 1.98, *p* = 0.02); in the standard group, there was no difference (HR 1.00, *p* = 0.998) (Fig. 1). After adjusting for age, sex, baseline SBP, LVEF, and AF type, baseline NT-proBNP ≥ 280 pg/mL was still a predictor in the aggressive cohort (HR 2.04, *p* = 0.0498) but not in the standard group (HR 0.82, *p* = 0.59).

Decrease in logNT-proBNP at 12 months compared to baseline was associated with decreased AF recurrence in the standard group (HR 0.69, *p* = 0.001), but not in the aggressive group (HR 0.89, *p* = 0.41).

Patients with baseline NT-proBNP ≥ 280 pg/mL had more additional (non-pulmonary vein) ablation (40.8% vs 19.2%, *p* = 0.005).

### Univariable predictors

Univariable predictors of recurrence in each group are shown in Table 2. Only baseline SBP was a predictor in the overall cohort. Predictors of recurrence in the aggressive group included baseline SBP (HR 1.03, *p* = 0.02), but not age (HR 0.99, *p* = 0.69). In the standard group, age (HR 1.03 *p* = 0.04) predicted outcome, but not SBP (HR = 1.01, *p* = 0.38).

### Multivariable analysis

In the whole study cohort, in multivariable analysis, logNT-proBNP (HR 1.03, 95%CI 0.84–1.27, *p* = 0.77), age (HR 1.02, 95%CI 0.99–1.05, *p* = 0.29), LVEF (HR 1.02, 95%CI 0.99–1.05, *p* = 0.19), AF type (HR 0.98, 95%CI 0.63–1.55, *p* = 0.94), sex (HR 1.04, 95%CI 0.6–1.80, *p* = 0.89) and baseline systolic pressure (HR 1.02, 95%CI 1.00–1.03, *p* = 0.08) were not predictors. Table 3 shows the multivariable predictors in each cohort. In the aggressive group, logNT-proBNP and baseline SBP were predictors. In the standard group, age was a predictor. NT-proBNP ≥ 280 pg/mL was also a predictor (adjusted HR 2.04, 95%CI 1.00–4.18**,** *p* = 0.0498) in the aggressive group, but not the standard group (adjusted HR 0.82, 95%CI 0.40–1.69, *p* = 0.59).

## Discussion

This study was a post-hoc analysis of the SMAC-AF, which randomized patients undergoing AF ablation to aggressive versus standard blood pressure management. We examined the association between NT-proBNP and AF outcomes. We found that in the overall study population, baseline logNT-proBNP was not associated with recurrence post-ablation. However, in patients who underwent aggressive BP control, after adjusting for baseline LVEF, SBP, age, and AF type, logNT-proBNP was associated with recurrence. We determined that a NT-proBNP concentration of above 280 pg/mL was associated with increased AF recurrence. This association was not found in the standard arm.

Our results indicate that in a population receiving AF ablation and aggressive BP control, NT-proBNP is a marker for recurrence; this association was not seen in those undergoing standard blood pressure control. NT-proBNP can be elevated by various mechanisms, including AF, LA and LV stretch [16]; it is known to increase with age, but not by isolated hypertension [8]. We did find a correlation between baseline NT-proBNP and type of AF, Age, and LVEF, but not with duration of AF, LA size, SBP. We also found that women had a higher baseline level of NT-proBNP, concordant with a recent study of biomarker differences between sexes [17].

Prior studies have shown association between pre-ablation NT-proBNP and recurrence [18], as well as in lone AF [14, 19]. However, we only found this in patients undergoing aggressive blood pressure control. This suggests that when hypertension is aggressively controlled to 120/80 mmHg, pre-ablation NT-proBNP becomes a more important marker of recurrence, indicating that other factors that play a role in determining AF recurrence; the mechanism of this is still unclear. Prior meta-analyses of NT-proBNP and AF recurrence have shown marked heterogeneity, suggesting that NT-proBNP may reflect several underlying factors [12, 13]. Left atrial stretch and remodeling, increased left ventricular filling pressures, and subclinical heart failure may all play a part in explaining this observation: higher NT-proBNP levels could reflect a more diseased substrate, dysfunctional hemodynamics, or diastolic dysfunction, which may have been less responsive to aggressive blood pressure lowering [20]. It has previously been shown that uncontrolled BP after ablation is associated with recurrence post-ablation [21]. Benefits of blood pressure control as upstream therapy for AF is thought to occur through substrate modification in the LA, and improved hemodynamics [22]. In our study, aggressive blood pressure control may have treated factors associated with hypertension, reducing their effect on recurrence, thus factors associated with high NT-proBNP became more important in predicting recurrence in this group. Meanwhile, in the standard treatment group, hypertension may still have had a stronger effect on recurrence.

A “J-curve” phenomenon has been described with BP lowering, where targeting levels too low results in harm [23, 24]**.** For patients being treated for hypertension, blood pressure under 120 systolic has been shown to be associated with a higher risk of AF [25]. Thus, there may be some patients with hypertension who benefit less from aggressive blood control. Our finding that higher NT-proBNP levels were only associated with recurrence in those undergoing aggressive BP control, suggests that higher NT-proBNP levels could help identify these patients so they could be targeted for additional screening and upstream therapy aimed at altering the atrial substrate to reduce recurrence of AF. Our finding that baseline SBP was associated with recurrence in the aggressive group (adjusted HR 1.03, *p* = 0.045) but not in the standard group (adjusted HR 1.01, *p* = 0.48) suggests that aggressive blood pressure control may be more beneficial in reducing AF recurrence in those with milder forms of hypertension.

The extent to which structural remodeling in AF is related to inflammation has been of significant interest. Elevation of CRP, interleukin-6 (IL-6), atrial and brain natriuretic peptide (ANP/NT-proBNP), and apelin in patients with AF suggests the presence of systemic inflammation in these patients; whether this is due to structural remodeling or not is unclear [7, 26–30]. Damage of atrial myocardium by repetitive rapid atrial activation may result in low grade inflammation which may further damage atrial myocardium resulting in further structural and electrophysiologic changes needed to maintain AF. Baseline CRP was not associated with recurrence in our study**.** Another example is a recently identified measure of collagen type I cross-linking and deposition that can reflect excessive atrial myocardial interstitial fibrosis [31]. Altered levels were shown to predict recurrence of AF post-ablation [32]. However, this novel biomarker is not widely available by common lab assays, and its clinical utility is unknown. These factors require further exploration to understand the effects that such processes may have on propagation and recurrence of AF post ablation. This may lead to personalization of therapies in patients that may have different underlying factors resulting in AF.

There are some limitations to our observations. The outcome of NT-proBNP was a prespecified secondary outcome; however, since this was a secondary analysis, and we did not correct for multiple testing, the usual limitations of a secondary analysis apply. The results should be considered hypothesis-generating, however raises the issue of whether or not patients with AF and hypertension have early or subclinical heart failure, and if this contributes to their symptoms and clinical outcomes; this will require further study. Not all patients (11.6%) in the cohort had a baseline NT-proBNP checked. The relatively few number of patients who had a low NT-proBNP level prior to ablation prevented us from investigating if the randomized intervention of aggressive blood control decreased recurrence in this population.

## Conclusion

In a hypertensive population undergoing AF ablation, baseline NT-proBNP levels predict recurrence in patients receiving aggressive blood pressure control, an association not seen in those receiving standard blood pressure control. This suggests that NT-proBNP levels may be useful as a biomarker to help select a subset of hypertensive patients who would benefit from aggressive blood pressure management, or other upstream therapies to target atrial substrate, to reduce AF recurrence post-ablation.

## Data Availability

Data will be made available upon reasonable request to the corresponding author.

## Abbreviations

AF: Atrial fibrillation
BP: Blood pressure
SBP: Systolic blood pressure
CRP: C-reactive protein
LVEF: Left ventricular ejection fraction
